# Whole genome based insights into the phylogeny and evolution of the Juglandaceae

**DOI:** 10.1186/s12862-021-01917-3

**Published:** 2021-10-21

**Authors:** Huijuan Zhou, Yiheng Hu, Aziz Ebrahimi, Peiliang Liu, Keith Woeste, Peng Zhao, Shuoxin Zhang

**Affiliations:** 1 College of Forestry, Northwest A&F University, Yangling, 712100 Shaanxi China; 2 Key Laboratory of Resource Biology and Biotechnology in Western China, Ministry of Education, College of Life Sciences, Northwest University, Xi’an, 710069 Shaanxi China; 3 USDA Forest Service Hardwood Tree Improvement and Regeneration Center (HTIRC), Department of Forestry and Natural Resources, Purdue University, 715 West State Street, West Lafayette, 47907 Indiana USA

**Keywords:** Diversification, Divergence time, Genome, Juglandaceae, Phylogenomics, Plastome

## Abstract

**Background:**

The walnut family (Juglandaceae) contains commercially important woody trees commonly called walnut, wingnut, pecan and hickory. Phylogenetic relationships and diversification within the Juglandaceae are classic and hot scientific topics that have been elucidated by recent fossil, morphological, molecular, and (paleo) environmental data. Further resolution of relationships among and within genera is still needed and can be achieved by analysis of the variation of chloroplast, mtDNA, and nuclear genomes.

**Results:**

We reconstructed the backbone phylogenetic relationships of Juglandaceae using organelle and nuclear genome data from 27 species. The divergence time of Juglandaceae was estimated to be 78.7 Mya. The major lineages diversified in warm and dry habitats during the mid-Paleocene and early Eocene. The plastid, mitochondrial, and nuclear phylogenetic analyses all revealed three subfamilies, i.e., Juglandoideae, Engelhardioideae, Rhoipteleoideae. Five genera of Juglandoideae were strongly supported. Juglandaceae were estimated to have originated during the late Cretaceous, while Juglandoideae were estimated to have originated during the Paleocene, with evidence for rapid diversification events during several glacial and geological periods. The phylogenetic analyses of organelle sequences and nuclear genome yielded highly supported incongruence positions for *J. cinerea*, *J. hopeiensis*, and *Platycarya strobilacea*. Winged fruit were the ancestral condition in the Juglandoideae, but adaptation to novel dispersal and regeneration regimes after the Cretaceous-Paleogene boundary led to the independent evolution of zoochory among several genera of the Juglandaceae.

**Conclusions:**

A fully resolved, strongly supported, time-calibrated phylogenetic tree of Juglandaceae can provide an important framework for studying classification, diversification, biogeography, and comparative genomics of plant lineages. Our addition of new, annotated whole chloroplast genomic sequences and identification of their variability informs the study of their evolution in walnuts (Juglandaceae).

**Supplementary Information:**

The online version contains supplementary material available at 10.1186/s12862-021-01917-3.

## Background

Phylogenomics applies genomic data to reconstruct the evolutionary biology of organisms [1–3], including the resolution of evolutionary relationships among and within family clades [4–7], genera, and closely related species [8–10]. Next generation sequencing (NGS) has made the generation of large-scale genomic data easier, cheaper, and greatly increased the availability complete chloroplast genomes [6, 9] and whole genome resequencing data [11]. The plastid genome has provided insight into molecular phylogeny and evolutionary relationships at many taxonomic levels [4, 9, 12, 13]. Foundational genetic studies of the Juglandaceae were based on analysis of selected loci [14–16]. Whole genome scale studies can be useful—and in some cases necessary—supplements to previous research. Whole genomes are particularly suited to resolution of evolutionary relationships where sequence variation is limited by taxonomic level, early divergence, large difference in morphology, rapid speciation or slow genome evolution [7, 17–20].

**Fig. 1:**
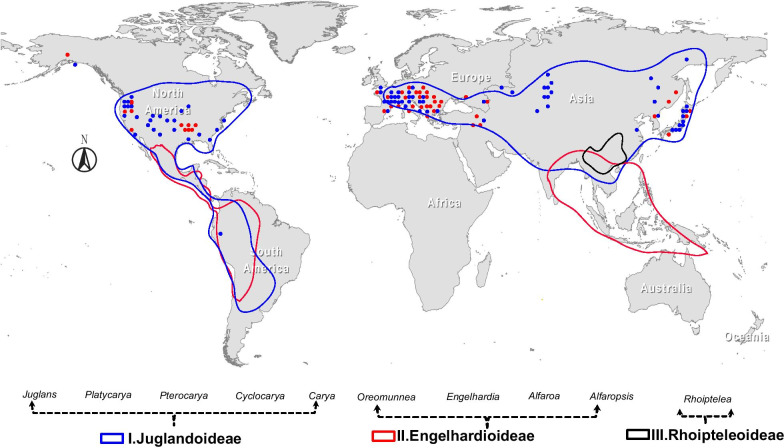
Geographic distribution of modern and fossil members of the Juglandaceae. Lined regions indicate the modern distribution of ten genera belong to the three subfamilies (Juglandoideae, blue line; Engelhardioideae-red line; and Rhoipteleoideae, black line). The map used ArcGIS (version 10.0). The source locations of Juglandaceae fossils used in our analyses are colored dots. blue-Juglandoideae (*Juglans*, *Platycarya*, *Pterocarya*, *Cyclocarya*, and *Carya*), red-Engelhardioideae (*Oreomunnea*, *Engelhardia*, *Alfaroa*, and *Alfaropsis*), and Black- Rhoipteleoideae (*Rhoiptelea*)

The walnut family (Juglandaceae) is distributed in both the Old and New World, from North and South America to southeastern Europe, eastern Asia, and southeastern Asia, from S10° to N49° [21–27] (Fig. 1). All species of Juglandaceae are perennial woody plants [28–32]. The accepted phylogeny for the Fagales shows the Juglandaceae is monophyletic and most closely related to the Myricaceae [28–30]. The Juglandaceae are lumped with five other families (Betulacea, Casuarinaceae, Fagaceae, Nothofagaceae, and Ticodendraceae) to constitute the order Fagales [28, 31, 33–38].

The Juglandaceae contains around ten extant genera (*Juglans*, *Pterocarya*, *Cyclocarya*, *Platycarya*, *Carya*, *Engelhardia*, *Alfaroa*, *Alfaropsis*, *Oreomunnea*, and *Rhoiptelea*) comprised of ca. ~ 60 total species [35–41]. Members of the family are considered some of the most important nut, medicinal, and timber trees. The phylogenetic relationships among and within genera of Juglandaceae are a complex puzzle that has been the subject of numerous studies [16, 24, 30, 31, 35–41]. Comparative morphology, i.e., primarily interpretation of the floral parts, was used to develop the classically accepted taxonomy and phylogeny of the family [21, 22, 39–45].

Although studies based on a limited number of loci (chloroplast DNA fragments) and fossil evidence have greatly advanced our understanding of Juglandaceae [16, 17, 23–27, 30, 31], some relationships within *Juglans*, *Carya*, and *Pterocarya* are weakly supported or conflicting; especially the relationship of *Platycarya* to *Carya*, and the position of *Cyclocarya* and *Pterocarya* in relation to *Juglans* [16, 36]. Other issues include the placement of the Rhoipteleaceae, a monotypic family containing only the species *Rhoiptelea chiliantha* [36, 43]. It was placed in the Juglandaceae by APG III (2009) system (Fig. 1) [44]. Similarly, the genus *Annamocarya* contains only one species, *A. sinensis*. Placement of *Annamocarya* within *Carya* is well-accepted [27, 35], although it shares a number of characteristics with walnuts (genus *Juglans*).

The evolution of the Juglandaceae remains a difficult problem too; hypothesized to have both ancient and recent extinctions and radiations [21, 27, 45], the family is considered species poor. The species that remain, however, are divergent in their ecology (wind versus animal-dispersed fruit) [31], and flower development [23].

The primary goal of this study was to increase the resolution of the molecular phylogeny of the Juglandaceae by maximizing the number of taxa sampled and the number of genetic markers used [23, 28, 31]. We selected 27 Juglandaceae taxa, slightly more than half of the ~ 60 recognized species from three subfamilies (Engelhardioideae, Juglandoideae, and Rhoipteleoideae), and from seven of the ten worldwide genera, making this the most comprehensive study to date. We used sequence data from matrilineally (chloroplast genomes and mitochondrial protein-coding genes) and biparentally (whole genome re-sequencing of nuclear genome SNPs) inherited DNA to illuminate the evolutionary history of the Juglandaceae. We also reanalyzed phylogenetic relationships of 55 species using ITS (Internal transcribed spacers) sequences. Our goal was to (1) reconstruct the phylogenetic relationships of the family Juglandaceae based on whole chloroplast genomes, whole genome re-sequencing of nuclear genome SNPs (nrSNPs), ITS, and sixteen mitochondrial protein-coding genes (mtCDS), with an eye toward the major unresolved systematic questions in this family, (2) compare the plastid genomes of Juglandaceae, and identify the location and extent of genetic variations in these genomes across within the Juglandaceae, (3) reconstruct a time-calibrated phylogeny of the Juglandaceae based on whole chloroplast genomes, (4) reveal the timing of diversification for important nodes within the family.

## Results

### Sequencing, assembly, and characteristics of Juglandaceae plastome

**Fig. 2:**
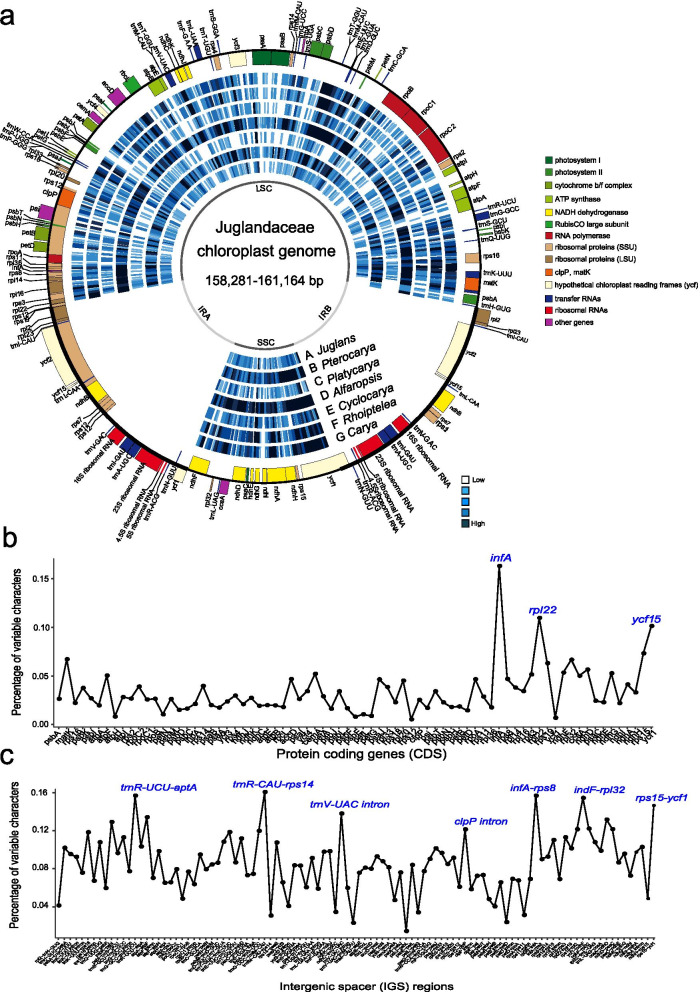
Variability of the family Juglandaceae represented over the circular map of *Juglans regia*, and comparison of percentage of variable characters in Juglandaceae plastomes. **a** Circular map comparing the chloroplast genomes of the genera of the walnut family (the reference chloroplast genome sequence NCBI accession number: KT963008; Hu et al. 2016a). The two inverted repeat regions (IRa and IRb) separate the large (LSC) and small (SSC) single copy regions, respectively. Genes represented by outside rectangles are on the positive strand, genes represented by inside rectangles are on the negative strand. Density of chloroplast SNPs is represented by a heatmap that varies from low (white) to high (dark blue). The circle depicts average SNP density estimated in 350 bp moving windows. *Carya* = *Carya cathayensis*, *Rhoiptelea = Rhoiptelea chiliantha*, *Alfaropsis = Alfaropsis roxburghiana*, *Platycarya = Platycarya strobilacea*, *Pterocarya = Pterocarya fraxinifolia*, *Juglans = Juglans ailantifolia.* Comparison of percentage of variable characters in Juglandaceae plastomes. **b** Protein-coding genes (CDS), **c** Intergenic spacer (IGS) regions. The peaks labeled in blue were highly variable genes or regions

All Juglandaceae plastomes were entirely syntenic, non-recombining circular genomes with conserved gene content and gene order (Table 1; Fig. 2a). The raw reads and sequence depth of Juglandaceae plastomes ranged from 29,975 to 13,878,699 bp and 0.19 to 86.54×, respectively (Additional file 1: Table S1). The Juglandaceae plastome has a mean length of 160,150 bp, and ranged from 158,281 bp (*Platycarya strobilacea*) to 160,585 (*Carya illinoinensis*) with four main parts; a large single-copy region (LSC; 87,900–90,477 bp), a small single-copy region (SSC; 18,296–18,533 bp), and two inverted repeat regions (IRs; 25,946–26,242 bp) (Table 1; Fig. 2a; Additional file 1: Table S1). The GC content of the Juglandaceae plastomes ranged from 36.1 to 36.3 % (Additional file 1: Table S1). The total number of annotated genes varied from 117 to 137, including 79–86 protein-coding genes (CDS), 30–40 tRNA genes (seven duplicated in the IRs) and 8 rRNA genes (four duplicated in the IRs) (Fig. 2a; Additional file 1: Table S1). Overall, a majority of the Juglandaceae plastomes encoded 134 genes (86 protein-coding genes, 40 tRNA genes and 8 rRNA genes) (Fig. 2a; Additional file 1: Table S1). There were four introns (*rpl2*, *rpl16*, *rps16*, and *rpoC1*) located in the IRs region and 13 introns in the LSC region in each of the plastomes (Fig. 2b, c). Seven tRNA genes, *trnI-CAU*, *trnL-CAA*, *trnV-GAC*, *trnI-GAU*, *trnA-UGC*, *trnR-ACG*, and *trnN-GUU* were duplicated and scattered in the inverted repeat (Fig. 2a). We aligned each of the protein-coding genes (CDS) of all species. Three potential pseudogenes (*infA*, *rpl22*, and *ycf15*) were identified, and their sequence verified using Sanger sequencing (Shagon Biotech, Shanghai, China) (Additional file 2: Fig. S1; Additional file 1: Table S2).

**Table 1:** Taxa and voucher information for plant material that provided Juglandaceae plastomes

Species	Total length	GC %	LSC	SSC	IR	Raw reads	Mapped reads	Sequencing Platform	GenBank No.
*Alfaropsis roxburghiana*	161,164	36	90,477	18,531	26,087	10,435,597	261,440	Illumina Hiseq2500	MH188300
*Carya cathayensis*	160,300	36.2	89,715	18,553	26,016	7,391,021	92,365	Illumina Hiseq2500	MH189594
*Carya hunanensis*	160,397	36.2	89,807	18,532	26,029	9,303,790	94,317	Illumina Hiseq2500	MH188303
*Carya illinoensis*	160,585	36.2	90,030	18,435	26,060	9,652,336	70,573	Illumina Hiseq2500	MH188302
*Carya kweichwensis*	159,780	36.3	89,264	18,430	26,043	9,087,431	236,036	Illumina Hiseq2500	MH188301
*Carya sinensis*	160,195	36.3	89,541	18,538	26,085	13,878,699	420,540	Illumina Hiseq2500	KX703001
*Cyclocarya paliurus*	160,562	36.1	90,007	18,477	26,039	9,073,816	277,193	Illumina Hiseq2500	KY246947
*Juglans ailantifolia*	160,353	36.1	89,931	18,376	26,023	12,918,979	145,907	Illumina Hiseq2500	MH188299
*Juglans cathayensis*	159,730	36.1	89,333	18,351	26,023	13,178,238	344,843	Illumina Hiseq2000	KX671976
*Juglans cinerea*	160,193	36.2	89,719	18,406	26,034	13,018,105	720,145	Illumina Hiseq2500	MH188298
*Juglans hindsii*	159,929	36.2	89,597	18,296	26,018	29,975	6,646	Roche 454	MH188297
*Juglans hopeiensis*	159,714	36.1	89,872	18,406	26,036	12,382,845	517,928	Illumina Hiseq2000	KX671977
*Juglans major*	160,221	36.1	89,766	18,372	26,034	9,407,594	513,705	Illumina Hiseq2500	MH188296
*Juglans mandshurica*	159,729	36.1	89,331	18,346	26,023	11,805,821	527,970	Illumina Hiseq2000	KX671975
*Juglans microcarpa*	160,065	36.2	89,637	18,383	26,022	85,030	22,781	Roche 454	MH188295
*Juglans nigra*	160,301	36.1	89,840	18,393	26,034	13,178,283	434,844	Illumina Hiseq2500	MH188294
*Juglans regia*	160,367	36.1	89,872	18,425	26,035	3,511,124	1,713,581	Illumina Miseq	KT963008
*Juglans sigillata*	160,350	36.1	89,872	18,406	26,036	12,225,897	402,317	Illumina Hiseq2000	KX424843
*Platycarya strobilacea*	158,281	36.1	87,990	18,399	25,946	12,345,252	79,584	Illumina Hiseq2500	MH189595
*Pterocarya delavayi*	160,174	36.2	89,640	18,510	26,012	9,295,098	179,137	Illumina Hiseq2500	MH188304
*Pterocarya fraxinifolia*	160,246	36.2	89,783	18,437	26,013	9,731,800	187,013	Illumina Hiseq2500	MH188291
*Pterocarya hupehensis*	159,770	36.2	89,229	18,505	26,018	9,605,591	273,616	Illumina Hiseq2500	MH188293
*Pterocarya insignis*	160,207	36.2	89,728	18,476	26,006	9,805,922	156,606	Illumina Hiseq2500	MH188292
*Pterocarya macroptera*	159,941	36.2	89,517	18,410	26,007	9,164,994	113,662	Illumina Hiseq2500	MH188290
*Pterocarya stenoptera*	160,202	36.2	89,727	18,433	26,021	11,542,884	186,936	Illumina Hiseq2500	MH188289
*Pterocarya tonkinensis*	160,096	36.2	89,600	18,482	26,007	7,449,017	160,611	Illumina Hiseq2500	MH188288

*LSC* large single copy, *SSC* small single copy, *IR* inverted repeat. Length of regions is given in number of base pairs (bp)

### Variation among walnut family based on chloroplast genome and nuclear sequencing data analysis

Comparison of the whole chloroplast genome sequences revealed a total of 18,050 SNPs and 2496 Indels (insertions and deletions), for a total of 6594 high-quality non-redundant variant positions, or approximately 5.66 SNPs/kb (Table 2; Fig. 2). A total of 4228 variant positions (64%) were found in intergenic regions. The remaining variants affected 88 genes, leaving 41 genes unaffected (Table 2). Several regions were remarkably variable, including *matK* (68.0 SNPs per kb), *ndhD* (56.5), *ndhF* (53.5), *rpoC2* (39.1), *rpoB* (26.5), *accD* (46.8), and *ycf1* (101.5). A total of 1,161,468 SNPs were identified from whole genome sequencing data (Additional file 1: Table S3) based on comparison with a *J. regia* reference genome [46]. The SNPs number, mapping ratio, heterozygosity, and heterozygosity ratio ranged from 202,314 to 1,143,008, 17.81% to 98.45%, 166 to 540,829, and 3.54% to 54.61%, respectively (Additional file 1: Table S3; Additional file 3: Fig. S2).

**Table 2:** Summary of variants from all Juglandaceae genomes based on comparison with *Juglans regia* whole genome sequences

Species	cp-SNPs	nr-SNPs	cp-Indels	Ts/Tv ratio	Mapped %	Heterozygosity	Homozygosity	Het-ratio %
*Alfaropsis roxburghiana*	1959	215,357	166	1.17/1.66	55.74	10,655	204,702	4.94
*Carya cathayensis*	1131	253,730	288	1.04/1.43	53.16	9,006	244,724	3.54
*Carya hunanensis*	1066	275,372	241	1.14/1.45	84.00	21,179	254,193	7.69
*Carya illinoensis*	1100	761,308	268	1.05/2.28	44.90	263,845	497,463	34.65
*Carya kweichwensis*	1102	326,824	24	1.09/1.43	66.91	23,680	303,144	7.24
*Carya sinensis*	1041	344,322	5	1.01/1.56	69.18	26,548	325,860	6.88
*Cyclocarya paliurus*	676	483,874	9	0.77/1.43	56.99	40,571	443,303	8.38
*Juglans ailantifolia*	433	443,059	134	1.02/1.61	37.64	21,704	421,355	4.89
*Juglans cathayensis*	458	1,143,008	23	1.05/1.61	45.01	120,312	1,022,696	10.52
*Juglans cinerea*	482	1,014,615	109	0.99/1.64	71.72	80,304	934,311	7.91
*Juglans hindsii*	376	361,913	46	1.12/1.43	68.84	17,962	343,951	4.96
*Juglans hopeiensis*	368	990,423	26	0.83/1.82	61.78	540,892	449,531	54.61
*Juglans major*	472	943,553	15	0.93/1.65	84.57	73,817	869,736	7.82
*Juglans mandshurica*	455	1,059,545	32	0.97/1.63	92.58	110,349	9,49,196	10.41
*Juglans microcarpa*	374	949,180	106	1.23/1.66	98.11	111,259	837,921	11.72
*Juglans regia*	0	742,382	0	0.00/2.29	96.69	225,592	516,790	30.38
*Juglans sigillata*	6	1,051,470	2	0.20/2.29	46.02	421,420	630,050	40.07
*Juglans nigra*	478	682,382	3	0.94/1.66	69.12	166	1871	8.14
*Platycarya strobilacea*	1465	319,852	22	1.33/1.49	97.71	39,263	280,589	12.27
*Pterocarya delavayi*	495	686,705	123	1.09/1.43	64.74	45,839	640,866	6.67
*Pterocarya fraxinifolia*	506	621,411	103	1.11/1.42	23.93	37,402	584,009	6.01
*Pterocarya hupehensis*	505	515,964	75	1.17/1.45	36.97	35,974	479,990	6.97
*Pterocarya insignis*	506	589,290	116	1.00/1.43	98.45	41,877	547,413	7.10
*Pterocarya macroptera*	513	430,744	153	1.11/1.42	72.96	23,897	406,847	5.54
*Pterocarya stenoptera*	483	653,976	28	1.12/1.39	86.64	53,662	600,314	8.20
*Pterocarya tonkinensis*	487	547,524	133	1.05/1.44	68.29	33,764	513,760	6.16
*Rhoiptelea chiliantha*	2126	202,314	179	1.21/1.71	58.22	11,023	196,822	4.78

*cp-SNPs* the number of SNPs of chloroplast genomes, *nr-SNPs* the number of SNPs of whole genome resequencing, *cp-Indels* the number of Indels of chloroplast genomes, *Ts/Tv ratio* the transition/transversion ratio based on chloroplast genomes and whole genome resequencing data respectively, *Mapped* the mapped ratio of whole genome resequencing data used common walnut genome sequence data, *Het-ratio* the Heterozygosity ratio of each samples based on the whole genome resequencing data

### Phylogenetic relationships of the Juglandaceae

**Fig. 3:**
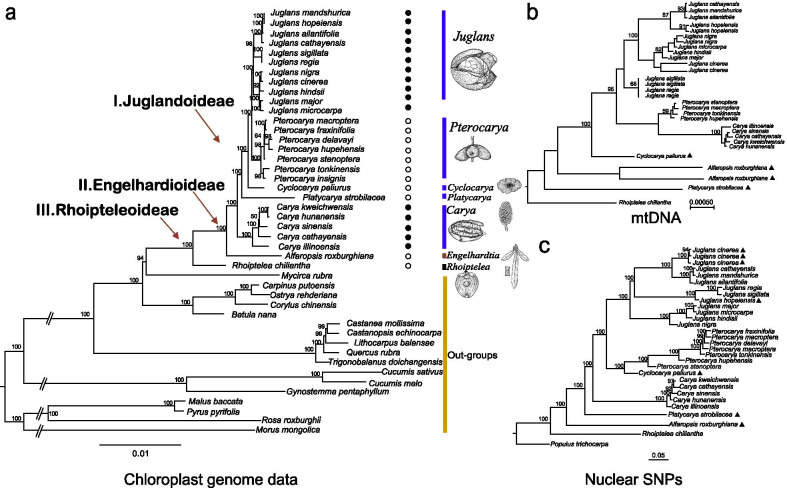
The Maximum Likelihood (ML) phylogenetic trees of Juglandaceae. Trees are based on **a** sixty one chloroplast protein-coding genes in the chloroplast, **b** 16 mtCDS fragement DNA sequence data, and **c** nuclear SNPs from whole genome resequencing data. For these trees, the PartitionFinder method for the best model combinations (Additional file 1: Table S4) was inferred by RAxML. Numbers at nodes correspond to ML bootstrap percentages (10,000 replicates). The three subfamilies are indicated with red arrows; Rhoipteleoideae (black bar), Engelhardioideae (dark red bar), and Juglandoideae (blue bar). Fruit morphology is shown using one species from each genus; the black solid circles indicate wingless fruits, hollow circles indicate winged fruits. Details for the outgroups (orange bar) are in Additional file 1: Table S1. The triangles indicate taxa with discordance between nuclear and chloroplast phylogeny

Based on best-fit partitioning schemes and models, the phylogenies returned from the RAxML and MrBayes analyses using 61 chloroplast protein-coding genes showed all branches highly supported (Fig. 3a). Within the Fagales, members of the Juglandaceae were closest to the Myricaceae and Betulaceae (Fig. 3b). Species within the Juglandaceae divided into three groups corresponding to the three previously described sub-families (Juglandoideae, Engelhardioideae, and Rhoipteleoideae) with 100% bootstrap (BS) support based on mtCDS and chloroplast genomes using maximum likelihood (ML) analysis (Fig. 3a, b).

Within the Juglandoideae subfamily, the species divided into five groups, corresponding to the five genera *Carya*, *Platycarya*, *Cyclocarya*, *Pterocarya*, and *Juglans* that were strongly supported as monophyletic (Fig. 3a). The genus *Pterocarya* was most closely related to *Juglans* (Fig. 3). The wheel wingnut (*Cyclocarya paliurus*) is the sole member of its genus in Juglandaceae. It was monophyletic and most closely related to *Pterocarya* based on chloroplast genomes (Fig. 3a). In *Carya*, Pecan (*C. illinoinensis* a North American species) was joined with the other four species of *Carya* (Asian hickories) with 100% BS. The cladograms supported the current division of *Carya* into two sections (Sect. *Sinocarya*, Asian hickories, i.e., *C. cathayensis*, *C. hunanensis*, *C. kweichowensis*, and *C. sinensis*; and Sect. *Apocarya*, which includes *C. illinoinensis*). We also confirmed that the genus *Annamocarya* (*A. sinensis*) is properly within *Carya* and closest to *Carya* Fig. 3) [27, 36–38]. The three sections within *Juglans* were well resolved with high bootstrap support (*J. regia* and *J. sigillata* into Sect. *Juglans/Dioscaryon*; *J. mandshurica*, *J. ailantifolia*, and *J. cathayensis* into Sect. *Cardiocaryon*; *J. cinerea*, *J. nigra*, *J. hindsii*, *J. microcarpa*, and *J. major* into Sect. *Trachycaryon* and Sect. *Rhysocaryon*) based on data from both chloroplasts and mitochondria (Fig. 3a, b). Branch lengths for *J. hopeiensis*/*J. mandshurica* and *J. regia*/*J. sigillata* were extremely short, further supporting their recent divergence.

Based on 1,161,468 nuclear SNPs, the phylogenetic analysis showed a generally well-supported clustering topology with high bootstrap values when rooted against *Populus trichocarpa* as the outgroup (Fig. 3c). The resulting phylogeny identified and provided 100 % support for the three sub-families that we observed in the genome-based phylogeny of the Juglandaceae (Fig. 3): Clade I (Rhoipteleoideae), clade II (Engelhardioideae), and clade III (Juglandoideae). Clade III (Juglandoideae) contained five genera *Platycarya*, *Carya*, *Cyclocarya*, *Pterocarya*, and *Juglans*, however, the relative placement of the three genera, *Carya*, *Platycarya*, and *Cyclocarya* was not consistent in the phylogenies based on the combined Cp and mitochondrial genomes versus the nuclear data. Although we only used one species in *Platycarya*, our results strongly supported the model that *Cyclocarya* and *Platycarya* are monophyletic with long branches and taxa-specific SNPs (Fig. 3c; Additional file 1: Table S3). Based on nuclear SNPs, we found a strong sister relationship of *Cyclocarya* to *Pterocarya* and, secondarily, to *Juglans* (Fig. 3c), as suggested by Manos et al. (2007) [17] and Larson-Johnson (2016) [35].

We reconstructed the Bayesian and ML trees based on ITS sequences of 55 Juglandaceae species (Fig. S3). The resulting phylogenetic tree showed that the three subfamilies, Juglandoideae, Engelhardioideae, and Rhoiptelioideae, cluster as monophyletic branches, however, support for the genera within the Juglandoideae was weak (< 50 %) (Additional file 4: Fig. S3). ITS alone produced cladograms markedly different than accepted topologies.

### The divergence time and historical diversification of Juglandaceae

**Fig. 4:**
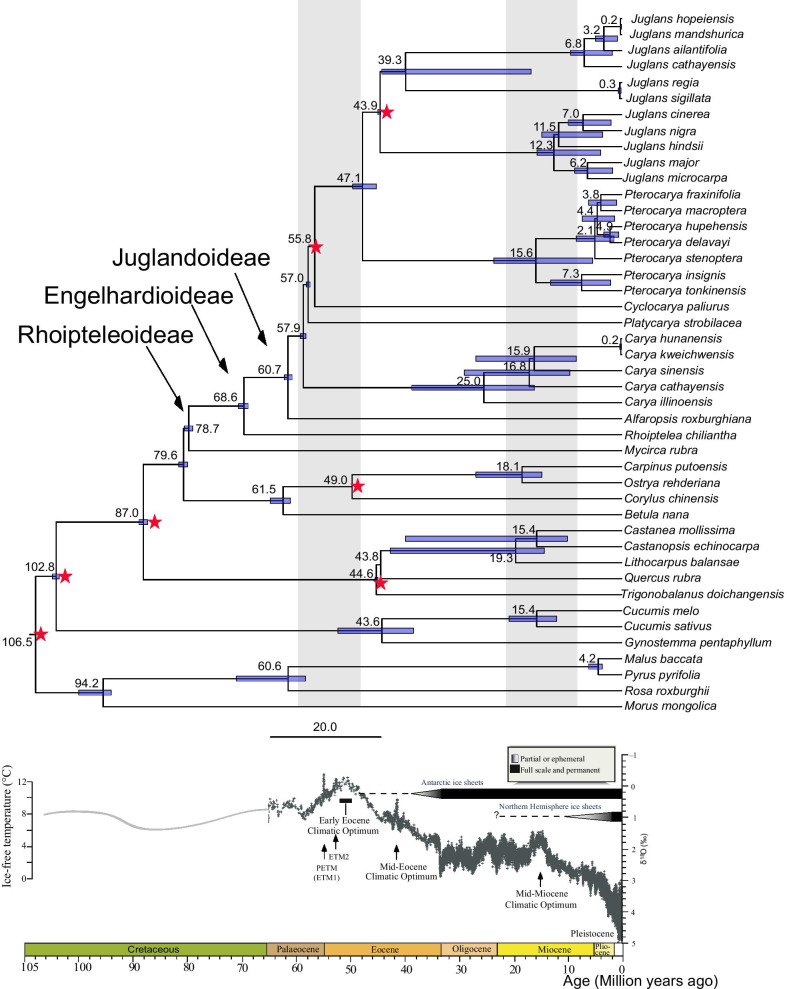
Time-calibrated phylogenetic tree of Juglandaceae based on 61 protein-coding genes (CDS) of chloroplast genomes. Mean divergence times estimated using a relaxed molecular clock model with 6 fossil priors (red stars). Blue bars across nodes indicate 95% HPD intervals around the mean divergence time estimates. Nodes are numbered as ages. The genera and subfamilies of Juglandaceae are shown in the figure and the geological time scale is shown below the tree. A stacked deep-sea benthic foraminiferal oxygen-isotope curve shows the evolution of global climate over the last 65 Mya, as modified from Zachos et al. (2001, 2008) [47, 48]. *PETM* Palaeocene–Eocene thermal maximum, *ETM* Eocene thermalmaximum, *Pl* pliocene. Reprinted by permission from Macmillan Publishers Ltd: Nature (451, 279–283), copyright (2008).

The stem age of Juglandaceae was estimated at 78.69 Mya (95% highest posterior density (HPD): 76.58–80.50 Mya). The walnut family diverged from the Myricaceae during the late Cretaceous (Fig. 4). During the Middle Cretaceous to Late Cretaceous, the three subfamilies Rhoiptelioideae, Engelhardoideae and Juglandoideae diverged at 68.64 Mya and 60.65 Mya (95 % HPD: 58.98–70.98 Mya), respectively. The crown age of the genus *Carya* was estimated at 57.88 Mya (95% HPD: 56. 67–60.32 Mya) during the Late Paleocene, *Platycarya* at 56.99 Mya (95% HPD: 56.80–58.80 Mya), and *Cyclocarya paliurus* at 55.80 Mya (95 % HPD: 54.30–57.30 Ma). The divergence of *Pterocarya* and *Juglans* was estimated at 47.10 Mya (95% HPD: 43.93–50.93 Mya) during the Early Eocene. Most genera of Juglandaceae diverged from 50.93 to 61.98 Mya in the relatively warm and dry climate of the Middle Paleocene to the Early Eocene (Fig. 4).

## Discussion

### Comparison of the genomes of the Juglandaceae

Both genome size and GC content among Juglandaceae plastomes were consistently more than the median genome size and GC content for land plant plastomes (Table 1). The nucleotide variability (Pi) across all 27 plastomes of Juglandaceae included in this study was 0.00791 (Fig. 2). Coding regions with the highest variation included *matK*, *atpI*, *rpoC2*, *rps14*, *aacD*, *psaI*, *ycf4*, *cemA*, *rpl33*, *infA*, *rps19*, *ndhF*, *rpl32*, *ndhD*, *ndhI*, and *ycf1*. Non-coding regions that were most variable were *matK*-*rps16*, *petN*-*psbD*, *ndhC*-*trn*V-UAC, *rbcL*-*psaI*, *psbE*-*petL*, and *rpl*14-*ycf*1. These regions of maximum variability will no doubt prove the most informative for phylogenetic studies in the Juglandaceae [6, 12]. Previous studies have identified *rpl22*, *rps19* and *ycf1* genes as the most variable genes in the Juglandaceae plastomes based on high indel density [12]. It was surprising, however, that the LSC region also contained variation, including 2577 bp differences among Juglandaceae plastomes, while SSC had 237 bp and IR had 296 bp differences among plastomes (Table 1). The identification of these regions of variability in protein-coding genes (CDS) and intergenic spacer (IGS) regions will be useful for the study of the evolution, phylogeny, biogeography of the walnut family (Juglandaceae) and Fagales [4, 27–29, 34, 35] and, potentially, for DNA barcoding. Three potential pseudogenes (*infA*, *rpl22*, and *ycf15*) will also be valuable genetic resource for study of plastid transfer to the nucleus and for studies of the evolution of the walnut family and Fagales [10, 20].

### Backbone relationship of Juglandaceae

The phylogeny of the Juglandaceae has been inferred based on microsporogenesis, morphology [22, 23], fossils [24, 27, 49], molecular markers [36–38], and combined data (morphology, fossils, and molecular data) [16, 27]. Several recent studies of phylogeny in the Juglandaceae have included data from plastomes [12, 36–39, 50–52]. The previously recognized subfamilies (Engelhardioideae and Juglandoideae), tribes (Platycaryeae and Juglandeae) and subtribes (Caryinae and Juglandinae) were all strongly supported [27, 31, 36–39]. Our phylogenetic analyses indicated that the Juglandaceae is subdivided into three major clades corresponding to the three subfamilies Rhoipteleoideae, Engelhardioideae, and Juglandoideae [17, 27, 28, 30, 35, 36, 51] (Fig. 1). The evidence for these three subfamilies can be found from morphology, fossil, and molecular data [17], fruits [23], and flower development [23]. The subfamily Engelhardioideae includes *Engelhardia*, *Oreomunnea*, and *Alfaroa* [23] (Fig. 1). Our results also supported the separation of *Alfaropsis* [17, 27] as a separate genus within Engelhardioideae (Additional file 4: Fig. S3). The Rhoipteleoideae (*Rhoiptelea chiliantha*) was a basal, monophyletic branch, which indicated that winged (dry) fruit was an ancestral character for the Juglandaceae (Fig. 3). The fruits of Myricaceae, the closest relative of the Juglandaceae, are small and fleshy, of a type common among Cretaceous flora [35–39]. The subfamily Rhoipteleoideae has only one species (*Rhoiptelea chiliantha*), which is a threatened and endemic in China [36–39, 43].

The subfamily Juglandoideae includes the commercially important nut-producing trees commonly called walnuts and butternuts (*Juglans*), pecan and hickory (*Carya*) [15, 27, 30, 36] (Fig. 1). The Persian walnut, *Juglans regia*, is one of the major nut crops of the world. Walnuts and hickories are also valuable timber trees [53]. Our plastid phylogenomic analyses fully resolved relationships among the major clades and genera of Juglandoideae (Fig. 3). Within subfamily Juglandoideae, four tribes are recognized (Juglandeae, Cyclocaryae, Platycaryae, and Hicorieae). Based on whole chloroplast genomes and sixteen mtCDS, the phylogenetic trees results strongly supported the previously published merger of the genera *Annamocarya* and *Carya* into the genus *Carya* (Fig. 3) [16, 27, 35–38]. Five genera, with their subgenera and sections were identified previously [24, 25, 27], i.e., *Carya* (here including *Annamocarya*), *Platycarya*, *Cyclocarya*, *Pterocarya*, and *Juglans*. These five genera resolved in our analysis with 100% support (Fig. 3). The phylogenetic relationships of the genera of the Juglandaceae reveal that *Carya* retains more primitive characters than *Platycarya* based on chloroplast DNA variation and morphology [54].

In previous studies, it was suggested the genus *Cyclocarya* is sister to genus *Platycarya* [17] based on fossil, chloroplast DNA fragments, and morphological data. Our data confirm this relationship (Figs. 3 and 4). Alternatively, it was suggested by Xiang et al. (2014) that *Platycarya* is sister to *Juglans* based on five chloroplast markers [31], and that *Carya* and *Platycarya* are sister groups [31]. Others considered *Cyclocarya* and *Juglans* to be sister groups [29]. Within Juglandoideae, our results strongly supported five genera (*Juglans*, *Pterocarya*, *Cyclocarya*, *Platycarya*, *Carya*) based on our chloroplast data (Fig. 3a), which is consistent with the phylogeny inferred from RAD-Seq data [36]. Using criteria based on fruit morphology, however, *Carya* and *Juglans* are sister groups [35], this relationship was not confirmed by our DNA-based analysis (Fig. 4), and *Cyclocarya* and *Pterocarya* are sister groups, a relationship supported in our data (Figs. 3 and 4) [35]. Previously, Smith and Doyle (1995) [54], based on chloroplast DNA and morphological data, concluded that *Platycarya* evolved earlier than *Carya;* our results based on nuclear resequencing (Fig. 3c) supported this conclusion. Our results based on sequencing the entire chloroplasts, however, indicated that the differentiation of *Carya* preceded *Platycarya* (Figs. 3 and 4; Additional file 4: Fig. S3), as suggested by Zhang et al. (2013) [30], although their differentiation, about 57 Mya, was roughly simultaneous. Many previous studies of Juglandaceae have suggested frequent hybridizations between species, which can prevent establishing conclusive taxonomies and bias the estimation of species divergence time. For instance, Zhang et al. (2019) [32] used whole genomic resequencing data to infer phylogenetic relationships and discover hybrid origins of species in *Juglans* [32]. Zhao et al. (2018) inferred walnut hybridized with a distinct lineage of *J. mandshurica* to form *J. hopeiensis*, a controversial taxon that results from phylogenomic and population genetic analyses, transcriptomics, Genotyping-By-Sequencing, and whole chloroplast genome data indicated is a horticultural variety [55].

### The phylogenetic relationships within genera of Juglandaceae

The phylogenetic relationships within genera of Juglandaceae were resolved partly in recent papers [27, 36–38], however, the species delimitations in *Carya*, *Pterocarya*, and *Juglans* remain a subject of debate. The generic circumscription of *Annamocarya* (also *C. sinensis*) has frequently been altered, and many genera have been segregated from or merged with *Carya* [27, 36, 37, 51, 56]. Our analyses fully supported some previously suggested intrageneric relationships, and added additional evidence supporting some of the recently altered generic circumscriptions based on analyses with more appropriate representation at the species level (Fig. 3; Additional file 4: Fig. S3) [27, 36–38]. Placement of the species *C. sinensis* (Chinese Hickory, beaked walnut, or beaked hickory) into *Carya* (Fig. 3; Additional file 4: Fig. S3) was well attested [27, 36, 37].

Species diversity centers of the genus *Pterocarya* occur in the northern temperate zone [27, 36, 37]. The previously unresolved intrageneric relationships of *Pterocarya* were resolved with high support using chloroplast genome data. *P. stenoptera*, *P. hupehensis*, and *P. tonkinensis* were clustered as a group (Fig. 3a); a second group consisted of *P. macroptera* and *P. fraxinifolia* (Fig. 3a) [37]. Morphology of the two groups within *Pterocarya* differs: group one species (*P. stenoptera*, *P. hupehensis*, and *P. tonkinensis*) have naked terminal buds, while the group two species *P. macroptera* and *P. fraxinifolia* have terminal buds with 2 to 4 caducous scales [37, 57]. We consider these taxa species relationships based on our chloroplast genome, mtDNA fragments, and nuclear SNPs data (Fig. 3, but see Fig. 4), however we did not complete a detailed phylogeny of *Pterocarya* because our sample pool was too small.

Our phylogenomic analyses resolved *Juglans* into three clear sections (*Cardiocaryon*, *Dioscaryon*, and *Rhysocaryon*) with high support (Fig. 3). Earlier phylogenies [22, 24] based on limited molecular data sometimes included a fourth section (*Trachycaryon*) containing only the North American species *J. cinerea*. The separation of *Trachycaryon* as distinct from section *Cardiocaryon* was inconsistent with morphology [21–24] and nuclear markers [15, 58], but congruent with fossil data [24] and the results of other analyses based on plastid sequences [12, 15]. In our phylogenetic analysis of nuclear genome SNPs, American butternut (*J. cinerea*) has high support (100 %) as sister to Section *Cardiocaryon* (Asian butternut, *J. cathayensis*, *J. mandshurica*, and *J. ailantifolia*) (Fig. 3c).

Based on sequence data from 16 mtCDS and 61 chloroplast protein-coding genes, our results supported the unification of *J. mandshurica*, *J. ailantifolia*, and *J. cathayensis* within sect. *Cardiocaryon* (Fig. 3b; Additional file 4: Fig. S3), consistent with a previous conclusion based on genotyping by sequencing data [22, 55]. We also confirmed that the Ma walnut (*J. hopeiensis*) arose from the resent hybridization of *J. regia* and *J. mandshurica* based on both matrilineal and biparental inheritance data (Fig. 3) [12, 55]. The placement of *J. cinerea* into *Rhysocaryon* (black walnuts) based on plastome sequence was clear (Fig. 3a), however, it belongs to *Cardiocaryon* (Asian butternuts) based on nuclear sequences (Fig. 3c), and its morphology is consistent with *Cardiocaryon* [12, 15]. In addition, *J. cinerea* can hybridize with members of *Cardiocaryon* and even *Dioscaryon*, but not with *Rhysocaryon* [59]. All other North American *Rhysocaryon* freely hybridize. The discordance between the *J. cinerea* nuclear genome and its plastome is almost certainly the result of a chloroplast capture [16, 32]. It is notable that the chloroplast of *J. cinerea* is not an ancient one (ancestral to the *Rhysocaryon*) but is instead most like *J. nigra* (Figs. 3 and 4). Our results indicated that the capture of a *Rhysocaryon* chloroplast by *J. cinerea* capture was relatively recent (Figs. 3 and 4). Hybridization and chloroplast capture between *Rhysocaryon* and *Cardiocaryon* apparently played a major role in the diversification of *Juglans*, as it did in other plant families [33, 36–38, 60]. The evaluation of divergence time using strictly bifurcating tree methods can be misleading because gene flow can result in underestimates of species divergence time [61].

### Dating the origin and historical diversification of Juglandaceae

Stem ages in the Juglandaceae are controversial [13, 17, 29, 30]. Most previous studies estimated a stem age of Juglandaceae about 84 Mya in the Cretaceous [25, 29], however, the divergence times for some genera remain uncertain [29, 30], as only a few studies have examined the divergence times of the major genera and within the species of the family [17, 30]. The lack of a robust phylogenetic framework and time tree has hindered development of a full understanding of the diversification of Juglandaceae.

The crown ages of Betulaceae, Myricaceae, and Casuarinaceae were 74.0 Mya (66.9–80.3), 90.4 Mya (85.0–94.6), and 82.8 Mya (74.7–88.6), respectively [31]. Estimates of the crown age of Juglandaceae varied among previous studies, 78 Mya by Manos et al. (2007) [17], 71 Mya by Larson-Johnson (2016) [36], 85.5 Mya by Sauquet et al. (2012) [29], 81.4 Mya by Mu et al. (2020) [36], 105 Mya by Zhang et al. (2021) [27], and 79.9 Mya by Xiang et al. (2014) [31]. Our results indicated the stem age of Juglandaceae to be during the late Cretaceous (78.58 Mya with 95% HPD: 76.58–80.50 Mya). The major diversification of the family is recorded in the pollen and megafossil record of the early Tertiary (~ 65.0 Mya) at the K-T boundary. The three subfamilies diverged during the Late Cretaceous to Early Palaeocene (60.7–68.6 Mya) (Figs. 3 and 4). Our estimates of divergence times among subfamilies and major genera were from 50.9 to 62.0 Mya in warm and dry habitats during the Middle Palaeocene to the Early Eocene (Fig. 4), which is largely consistent with the estimates of Xiang et al. (2014) [31], Larson-Johnson (2016) [35], and Zhang et al. (2021) based on fossil, morphological, molecular, and (paleo) environmental data [27]. We estimated the divergence time of *Juglans* and *Pterocarya* to have been ~ 47 Mya (Fig. 4; Manos et al. 2007, ~ 55 Mya) [17]; *Pterocarya* and *Cyclocarya* diverged ~ 56 Mya (Fig. 4; Manos et al. 2007, ~ 59 Mya [17], Zhang et al. 2021, ~ 50 Mya [27], and Mu et al. 2020, ~ 60 Mya [36]). Three groups [Xiang et al. (2014), Larson-Johnson (2016), and Song et al. (2020)] estimated a divergence time between *Juglans* and *Pterocarya* of ~ 24 Mya [31, 35, 37], and ~ 18 Mya between *Pterocarya* and *Cyclocarya* [35]. By the end of the Eocene, *Cyclocarya* and *Platycarya* became extinct in North America but survived in Eurasia [25]. Our results indicated *Carya* emerged as an animal-dispersed genus about 58 Mya, considerably earlier than the estimate (~ 44 Mya) of Larson-Johnson (2016) [35] and Song et al. (2020, ~ 40 Mya) [37], but later than the estimate (~ 80 Mya) of Zhang et al. (2021) [27], although we agree that the overwhelming majority of winged and wingless fruited *genera* diverged or diversified during the Paleogene, probably reflecting adaptation to changing regeneration regimes [62]. We estimated the divergence time between the Juglandoideae and Engelhardioideae, which are reciprocal monophyly subfamilies, was ~ 68.6 Mya, later than the estimate of Mu et al. (2020) was ~ 79.18 Mya [36].

From the early Tertiary to the Neogene there was likely extensive migration and exchange among North Atlantic, North America, western European, and Asian flora [25]. Interestingly, most species within the extant genera diversified between 18.5 and 8.5 Mya in warm and dry environments of the Early Miocene (Fig. 4), a period of especially rapid speciation within *Juglans* and *Pterocarya*. Juglandaceae species diversity in from Oligocene to Pliocene with a rapid increase elucidated by Zhang et al. 2021 (between 30 and 5 Mya) [27], Mu et al. 2020 (between 20 and 5 Mya) [36], and Song et al. 2020 (between 13 and 5 Mya) [37]. Some closely related taxa within *Juglans* appear to have diverged relatively recently, under the influence of climate change during the Quaternary glacial period (Fig. 4; Bai et al. 2017) [63]. For example, *J. regia* and *J. sigillata*, *J. mandshurica* and *J. hopeiensis*, and *Carya hunanesis* and *C. kweichwensis* (Fig. 4). Overall, the Juglandaceae reflect a complex evolutionary history and diversification affected by changes in geography, distinctive distributions, climate changes, coevolution with animals. Biotic interactions (e.g., pathogens) no doubt also had a role in driving species abundance and distribution [63], but biotic interactions of that type are difficult to detect from current data [36–39].

## Conclusions

Our results are a first attempt to use whole genomes to elucidate the characterize sequence divergence and evolutionary history in the Juglandaceae. Evidence of early lineage diversification, hybridization and extinction lead us to predict complex evolutionary histories for the extant species in the Juglandaceae. A fully resolved, strongly supported, time-calibrated phylogenetic tree of Juglandaceae will provide an important framework for studying classification, diversification, biogeography, phenotypic evolution, gene function and comparative genomics of this important family. Our results supported some recently clarified circumscriptions of controversial genera, although our taxonomic sampling is insufficient to stand alone as definitive. Variation within our newly annotated whole chloroplast genomic sequences (available in GenBank) should be a useful resource for study of the evolution, for DNA barcoding, phylogeny, biogeography, and studies of genetic variation in the walnut family (Juglandaceae). Wider plastid phylogenomics, whole genomes (nuclear data), a more complete fossil record, better dating of the fossil record, and more studies of morphology will all be needed to fully reconstruct the phylogeny of woody plant families such as the Juglandaceae and other families of Fagales.

## Methods

### Taxon sampling, genomic DNA extractions, library, and sequencing

We analyzed 27 species of Juglandaceae from seven genera that span the taxonomic, geographic, and morphological range of the family. These were contextualized using published plastomes of nine species of Fagales (include four species for Betulaceae, and five species for Fagaceae), three species of Cucurbitales, and four species of Rosales (Additional file 1: Table S1). The voucher specimens were deposited in the herbarium of Key Laboratory of Resource Biology and Biotechnology in Western China (Ministry of Education), Northwest University (Table 1). We collected fresh leaf samples from the field, and the samples were stored in air tight bags filled with silica gel desiccant for later DNA extraction.

Total genomic DNA was extracted from 200 mg of silica gel-dried leaves using a modified CTAB (cetrimonium bromide) method [64, 65]. The DNA concentration was quantified using a NanoDrop spectrophotometer (Thermo Scientific, Carlsbad, CA, USA). A paired-end (PE) library with 350 bp insert size was constructed using the Illumina PE DNA library kit according to the manufacturer’s instructions and sequenced using an Illumina Hiseq2500 by Novogene (www.novogene.com, China).

### Mitochondrion protein-coding genes (mtCDS) primer design and PCR amplification

We investigated genetic variation within mitochondrial protein-coding genes (mtCDS) to evaluate phylogenetic relationships of the Juglandaceae. We used a total of sixteen primers designed from the complete mitochondrion sequence of *Populus tremula* (NCBI accession number: KT337313.1) using Primer3 (Sangon Biotech in Shanghai, China). Primers were targeted to the sequence of mitochondrial protein-coding genes of *P. tremula* (Additional file 1: Table S5). PCR amplification was carried out on a SimpliAmp Thermal Cycler (Applied Biosystem, USA) in 20 µL reaction volumes (10 µL 2 × PCR Master Mix including 0.1 U Taq polymerase/µL; 500 µM each dNTP; 20 mMTris-HCl (pH8.3); 100 mMKCl; 3.0mM MgCl_2_ (Tiangen, Beijing, China),0.5 µL each primer, 2 µL BSA, 2 µL of 10 ng/µL DNA). The PCR was programmed for 3 min at 94 °C followed by 35 cycles of 15 s at 93 °C, 1 min at annealing temperature (Additional file 1: Table S5), 30 s at 72 °C and extension of 10 min at 72 °C. After PCR amplification, fragments were sequenced by Sangon Biotech (Shanghai, China).

### Plastomes assembly and annotation

The sequenced and assembled plastomes were quality controlled using the NGSQC toolkit v2.3.3 trim tool to remove low quality reads, unknown bases, adapter sequences, and sequencing errors [66]. Short reads were assembled into long contigs using SPAdes Genomic Assembler v3.6.0 [67], followed by manual checking and finishing. We used a reference *J. regia* complete chloroplast genome (Genbank accession number KT963008) in this study [50]. The contigs were assembled in Geneious v8.0.2 [68]. To exclude nuclear DNA, we used BLAST to remove contigs that did not align to a reference plastome from *J. regia* [50]. A reference-based assembly allowed us to reconstruct each of all other species [13].

After we identified the boundaries between the inverted repeats (IR) and the single copy regions, i.e., the Large Single Copy (LSC) and Small Single Copy (SSC) regions, the completed plastomes were annotated using the online software DOGMA based on the *J. regia* reference [50, 68, 69]. We manually annotated start and stop codons and other regions of interest using Geneious v8.0.2 [50]. A circular representation of each plastome was visualized in OGDraw [70]. Finally, gene content, order, and variability were analyzed in Geneious and R [71]. The plastid genomes data were deposited in National Center for Biotechnology Information (NCBI), the accession numbers were KX703001 to KX703038 (Table 1).

### Variant calling

Using paired-end (2 × 150 bp) Illumina sequencing, we obtained high sequencing depth (> 30×) per sample based on alignment with the *J. regia* reference plastome [50]. After aligning the re-sequenced reads, we processed the alignments to remove duplicate reads and applied a series of quality control filters with the intent of limiting false-positive variants. Sequence reads passing Illumina’s quality control filter were aligned using bwa-mem algorithm of BWA v0.7.12 [72] and then mapped to the *J. regia* plastid genome. Only uniquely mapped reads were retained, which removed the repeat region IR. Duplicate reads were removed from individual sample alignments using Picard tools v2.5.0 (https://github.com/broadinstitute/picard) Mark Duplicates function and assigned genomic positions for each accession based on the alignment files generated by SAMtools v0.1.19 [73].

The SNPs (single nucleotide polymorphisms) and small Indels (insertion-deletion) among Juglandaceae plastid genome accessions were identified if they were supported by at least three mapped reads. Following bwa-mem mapping, the rest of the sequencing pipeline was performed using the toolkit GATK v3.5.0 [74]. Reads present in areas surrounding Indels were realigned using the built-in function Indel Realigner, after which SNPs were called using Unified Genotyper. Finally, a series of quality filters were applied to reduce systematic errors, including quality-by-depth ratio (QD) < 10, ReadPosRankSum < − 8.0, depth coverage (DP) ≥ 30, probability of strand bias (FS) > 10.0, SNPs that passed these filters were kept for subsequent analyses. Finally, we use the stats module in the bcftools v1.1 to count SNPs and Indels and calculate Ts/Tv (transition/transversion) rates [75].

In this study, we called the nuclear SNPs from all samples of Juglandaceae (Additional file 1: Table S3). The Illumina paired-end reads from each sample were first processed to remove adaptor and low-quality sequences using Trimmomatic [76]. The cleaned unique reads were aligned to the common walnut reference genome version 1.0 (https://treegenesdb.org/FTP/Genomes/Jure/) using BWA [46, 73], and only uniquely mapped reads were retained. Following mapping, genotypes were assigned to each genomic position for each sample based on the alignment files generated by SAMtools [72]. Single nucleotide polymorphism (SNPs) and small indels (insertion and deletion) in the 27 samples were identified using GATK [74]. The redundant reads were then filtered based on the location of clean reads in the reference genome using software Picard (Picard: http://sourceforge.net/projects/picard/). We used GATK’s Haploype Caller (local haplotype assembly) algorithm for SNPs and InDels based on each sample.

### Partition strategy and phylogenetic analysis

To infer the evolutionary relationships among the 27 Juglandaceae plastomes, and to test the phylogenetic signal from different regions of the plastomes, we reconstructed the Juglandaceae phylogeny using the following four datasets based on the exons of protein-coding genes, whole chloroplast genome data, mitochondrial protein-coding genes (mtCDS), whole genome re-sequencing of nuclear genome SNPs (nrSNPs), and ITS (Internal transcribed spacers) sequences; to avoid large amounts of missing data in the phylogenetic analyses, sixty-one protein coding genes that were shared by all 44 taxa were extracted and aligned (Additional file 1: Table S4). Best-fit partitioning schemes and models were selected using the greedy search mode implemented in PartitionFinder v2.1.1 [77] (Additional file 1: Table S6).

Plastomes were aligned using default settings in MAFFT v7.245 [78]. The resulting alignments were manually checked in Geneious v8.0.2 [50]. The best-fit nucleotide substitution model for all our plastome data sets was determined (as suggested by Modeltest v3.7 with the Akaike information criterion (AIC) [79, 80]. A concatenated data set was analyzed using Bayesian Inference (BI) and Maximum Likelihood (ML) analysis in MrBayes v3.2.6 [53] or RAxML v8.1.24 [81]. BI trees were produced by MrBayes v3.2.6 set at 10,000,000 generations. Two independent Markov chain Monte Carlo (MCMC) chains were run, each with one cold chain and three incrementally heated chains. Trees were sampled every 10,000 generations, with the first 25 % of the trees discarded as burn-in. Stationarity was considered reached when the average standard deviation of split frequencies was < 0.01. The Maximum Likelihood (ML) trees were generated using RAxML v8.1.24 using a GTRGAMMA model [81]. For ML analysis, difference general time reversible models were performed with all data sets. For all analyses, 10 independent ML searches were conducted, bootstrap support was estimated with 1000 bootstrap replicates, and bootstrap (BS) proportions were drawn on the tree with highest likelihood score from the 10 independent searches. We generated multiple mtCDS sequence alignments using ClustalX with default parameters [82]. The phylogenetic tree analysis was performed using MEGA7 [83].

For the phylogenetic tree analysis based on nuclear genome data, we selected a total of 1,161,468 SNPs with minor allele frequency (MAF) ≥ 5 % and missing rate per site ≤ 10 % for phylogenetic analyses. A Maximum Likelihood (ML) tree was constructed using RAxML v8.1.24 in 1000 bootstrap replicates [81]. To gain a better understanding of the species relationships, we selected 55 species to represent all extant genera in the Juglandaceae for which internal transcribed spacer (ITS) sequence data are available in NCBI (Additional file 1: Table S7). We generated multiple ITS sequence alignments using ClustalX with default parameters [82], and a phylogenetic tree analysis using Maximum Likelihood analysis [81].

### Divergence-time estimation and fossil calibration

We estimated the divergence time of Juglandaceae species based on complete chloroplast genome data combined with six fossil calibrations (Additional file 1: Table S8) [24, 25, 29]. Penalized likelihood (PL) dating analyses were conducted using the treePL v1.0 program [84]. To identify the appropriate level of rate heterogeneity in the phylogram, a data-driven cross-validation analysis was conducted with treePL v1.0. One thousand bootstrap replicates with branch lengths were also generated using RAxML v8.1.24 for calculating the confidence age intervals with TreeAnnotator as implemented in BEAST v2.4.5 with a GTR + I + G substitution model and an uncorrelated lognormal relaxed-clock [85, 86]. The phylogenetic trees were then compiled into a maximum clade credibility tree using Tree Annotator v1.8.0 [87]. The program FigTree v1.3.1 (http://tree.bio.ed.ac.uk) was used to visualize mean node ages and highest posterior density (HPD) intervals at 95 % (upper and lower) for each node and to estimate branch lengths and divergence times.

## Supplementary Information

**Additional file 1: Table S1.** The taxa, Genbank accession number, and family of 41 species used for phylogenetic analysis. **Table S2**. Characterization of three primers to verified three pseudogenes. **Table S3.** Summary of variants from all Juglandaceae genome resequencing based on comparison with *Juglans regia* whole genome sequences. **Table S4.** Protein-coding genes (n = 61) included in the phylogenetic analysis. **Table S5.** Characterization of sixteen mitochondrion protein-coding genes (mtCDS) primers used in this study. **Table S6.** The PartitionFinder method for the best model combinations of the ML phylogenetic tree based on 61 protein-coding genes. **Table S7.** Sources of Internal transcribed spacer (ITS) sequences used in this study. **Table S8.** Fossil evidence used for estimation divergence time of Juglandaceae.

**Additional file 2: Fig. S1.** Alignment of three pseudogenes in the all Juglandaceae species and five eudicot outgroup plastomes. (a) *infA*. (b) *rpl22*, and (c) *ycf15*. The black box with an asterisk represents stop codons. (d) The PCR amplication products of three pseudogenes. Their identity was verfied by Sanger sequencing (primers see Table S8). (e) The amino acid sequence of three pseudogenes of ten species of the Juglandaceae.

**Additional file 3: Fig. S2.** The properly mapped ratio (red line) and heterozygosity ratio (blue line) of whole genome sequence data from Juglandaceae. All comparisons are to *Juglans regia*.

**Additional file 4: Fig. S3.** The Maximum Likelihood (ML) phylogenetic tree of 55 Juglandaceae species based on ITS (Internal transcribed spacers) sequences inferred by RAxML. Data from NCBI, see Additional file 1: Table S6 for details. Numbers at nodes correspond to ML bootstrap percentages (10,000 replicates). The three subfamilies of the Juglandaceae are indicated with shading: Rhoipteleoideae [grey (C)], Engelhardioideae [red (B)], and Juglandoideae [blue (A)] are shown.

## Data Availability

The annotated newly chloroplast genomic sequence were deposited into GenBank (MH188288-MH188304, MH189594-MH189595; Details see Table 1).
