# Supplementary figures and images for: Whole genome based insights into the phylogeny and evolution of the Juglandaceae
Source: BMC Ecol Evol. 2021 Oct 21;21:191. doi: 10.1186/s12862-021-01917-3 (PMC8529855; doi:10.1186/s12862-021-01917-3)

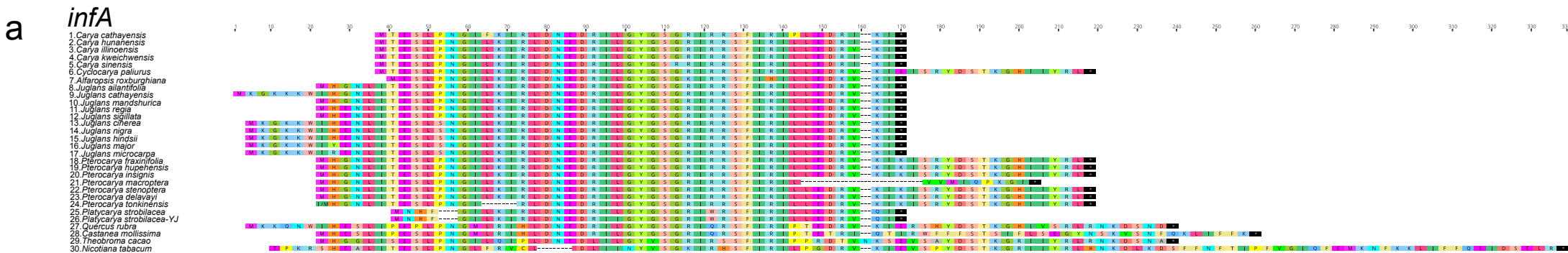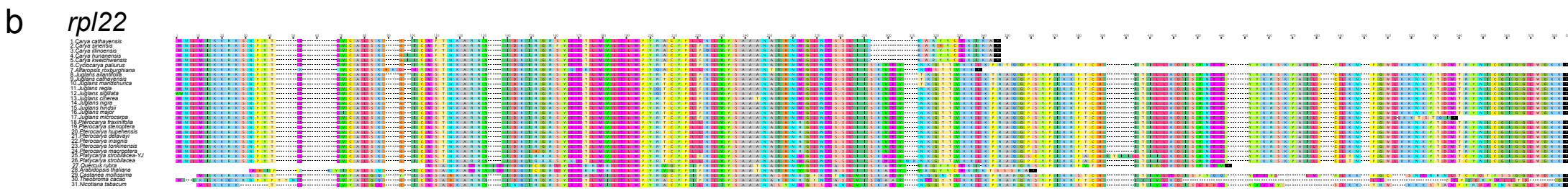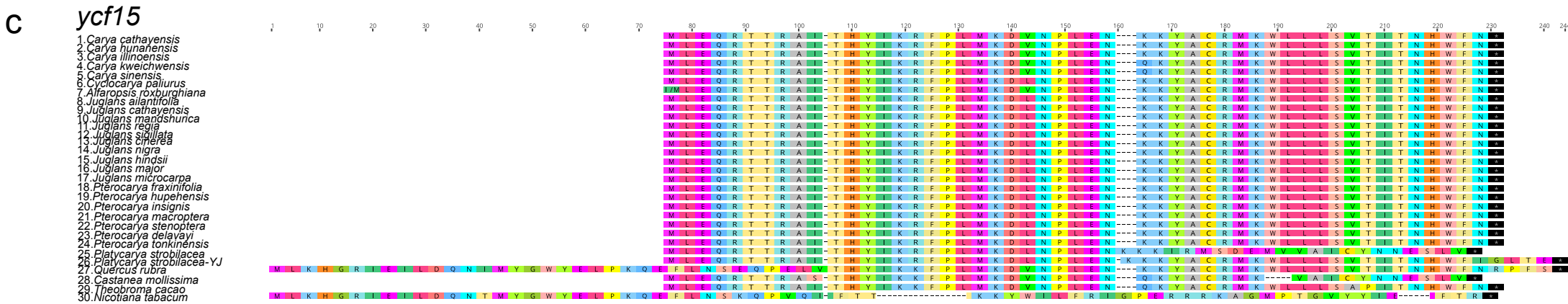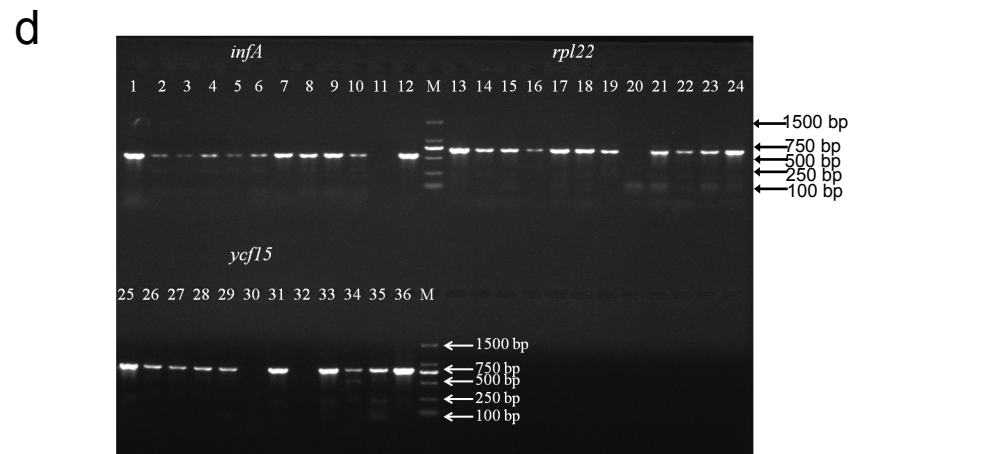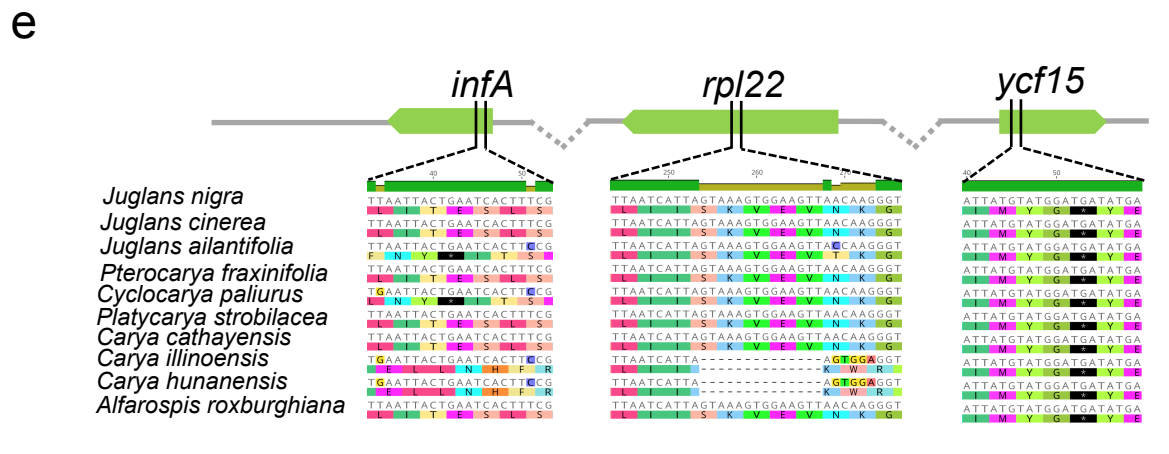

Supplement: Supplementary file 2 — Additional file 2: Fig. S1. Alignment of three pseudogenes in the all Juglandaceae species and five eudicot outgroup plastomes. (a) infA. (b) rpl22, and (c) ycf15. The black box with an asterisk represents stop codons. (d) The PCR amplication products of three pseudogenes. Their identity was verfied by Sanger sequencing (primers see Table S8). (e) The amino acid sequence of three pseudogenes of ten species of the Juglandaceae. [file 12862_2021_1917_MOESM2_ESM.pdf]

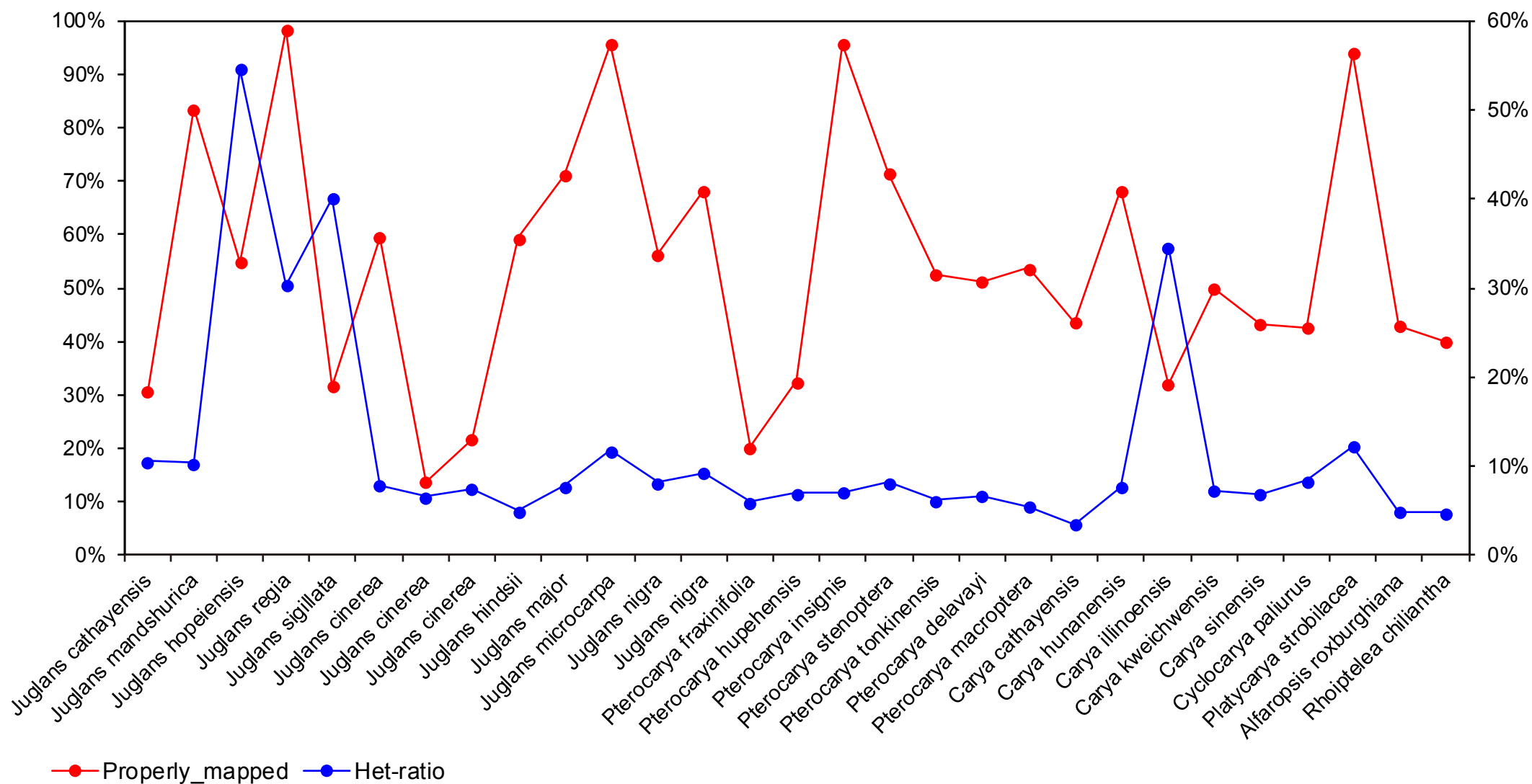

Supplement: Supplementary file 3 — Additional file 3: Fig. S2. The properly mapped ratio (red line) and heterozygosity ratio (blue line) of whole genome sequence data from Juglandaceae. All comparisons are to Juglans regia. [file 12862_2021_1917_MOESM3_ESM.pdf]

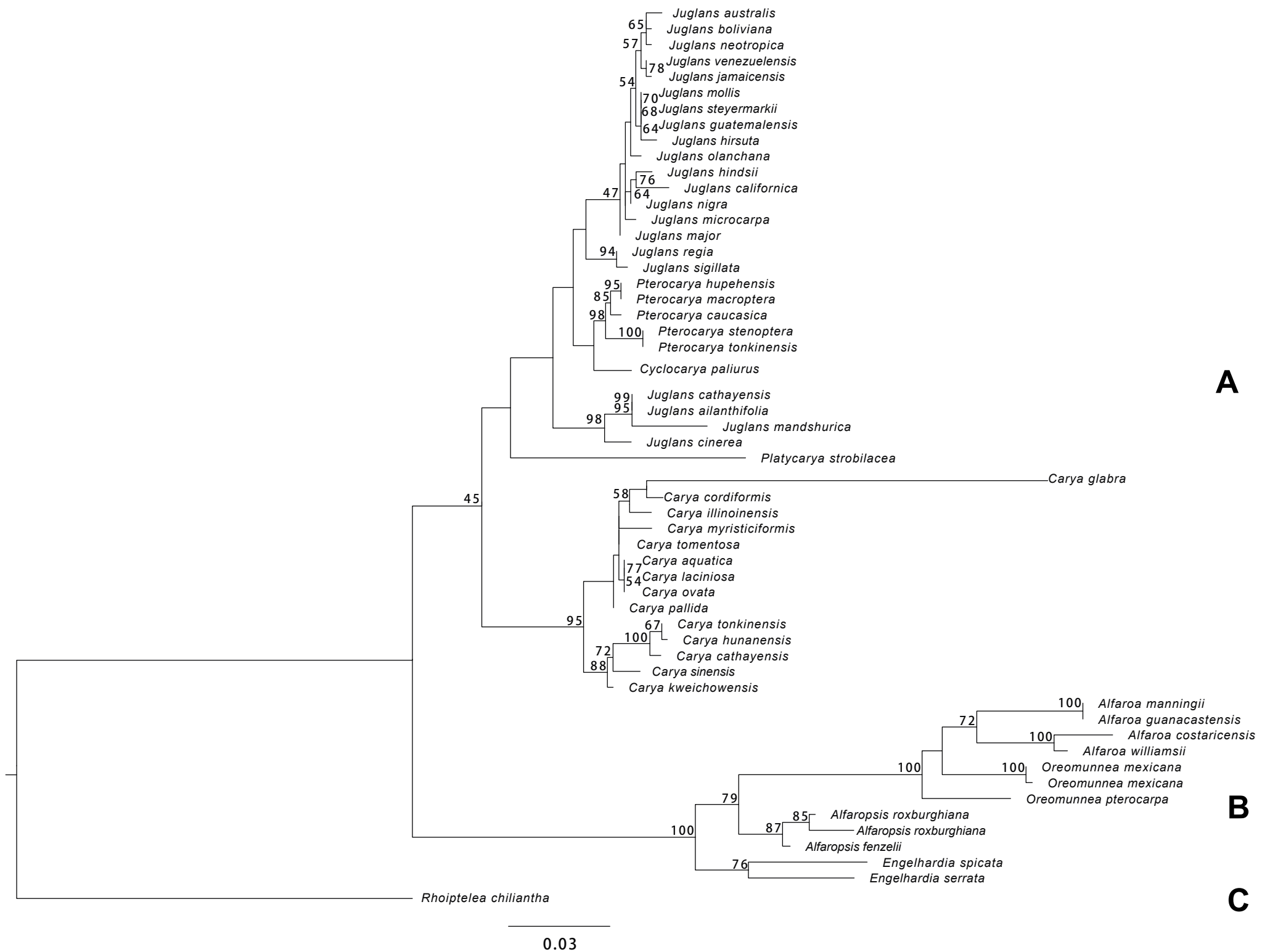

Supplement: Supplementary file 4 — Additional file 4: Fig. S3. The Maximum Likelihood (ML) phylogenetic tree of 55 Juglandaceae species based on ITS (Internal transcribed spacers) sequences inferred by RAxML. Data from NCBI, see Additional file 1: Table S6 for details. Numbers at nodes correspond to ML bootstrap percentages (10,000 replicates). The three subfamilies of the Juglandaceae are indicated with shading: Rhoipteleoideae [grey (C)], Engelhardioideae [red (B)], and Juglandoideae [blue (A)] are shown. [file 12862_2021_1917_MOESM4_ESM.pdf]
